# Best of Both Worlds: Comment on "(Re) Making the Procrustean Bed? Standardization and Customization as Competing Logics in Healthcare"

**DOI:** 10.15171/ijhpm.2017.99

**Published:** 2017-08-16

**Authors:** Catherine Needham

**Affiliations:** Health Services Management Centre, University of Birmingham, Birmingham, UK.

**Keywords:** Standardization, Customization, Personalization, Phronesis

## Abstract

This article builds on Mannion and Exworthy’s account of the tensions between standardization and customization within health services to explore why these tensions exist. It highlights the limitations of explanations which root them in an expression of managerialism versus professionalism and suggests that each logic is embedded in a set of ontological, epistemological and moral commitments which are held in tension. At the front line of care delivery, people cannot resolve these tensions but must navigate and negotiate them. The legitimacy of a health system depends on its ability to deliver the ‘best of both worlds’ to citizens, offering the reassurance of sameness and the dignity of difference.

Russell Mannion and Mark Exworthy’s article [“(Re) Making the Procrustean Bed? Standardization and Customization as Competing Logics in Healthcare”] draws attention to the tension between standardization and customization as ‘a critical fault-line within many health systems.’^1^ Examples of both abound. The evidence-based medicine movement, they argue, has created an environment in which standardized interventions seek to remove unwanted variation in diagnosis and treatment; surgical procedures become dominated by protocols and checklists; audit processes ensure that risk is managed by adherence to rules. At the same time personalized medicine allows more tailored intervention; patients are becoming active choice agents and partners in co-productive efforts to shape their own care and even to manage their own health budget. The authors argue that standardization and customization can best be understood as rival institutional logics which impact on professional practice and patient care. In explaining the ways in which these themes are evident in everyday practice, the authors present them as sedimented logics, layered upon each other, which sometimes take hybrid forms (eg, ‘mass customization’). Through better understanding these logics, they suggest, we can make sense of how they play out at the meso (organizational) and micro levels (team and individual).

In drawing attention to the tensions and contradictions of these two dominant logics within health systems, their article very helpfully highlights the dilemmas which are faced by staff and patients encountering these tensions. What I seek to do here is to pick up the rather neglected point in the original article of why these two logics are so omnipresent in health systems. On the one hand this may seem a fruitless endeavour: a better question might be why would large and complex systems have only one institutional logic. All large systems draw on and hybridise multiple logics, and only a die-hard believer in rationalist explanations could be surprised that something as intricate as a health system would contain within it multiple overlapping and contradictory logics. Even the two logics themselves are not internally consistent (for example, customisation at times means transferring choice and risk to the self-determining patient-consumer; at other times it means clinicians working co-productively with patients, taking a whole person approach and shared decision-making).

However exploring explanations for the co-existence of the two apparently conflicting sets of principles (standardization and customization) tells us something interesting about how health systems are evolving and the social context within which they are located. Four explanations are considered here: the first relates to the clash between managerialism and professionalism; the second is an epistemological explanation about knowledge and authority; the third relates to a functional distinction about what health systems do; and the fourth locates the tension in an essential moral tension between sameness and difference.

The first explanation and the one offered by Mannion and Exworthy is that this is a clash between managerialism and professionalism. Managers prize efficiency and the erasure of risk, leading them to embrace standardisation; clinicians like other professionals want to exercise discretion and expert judgement rather than adhering to rules. Mannion and Exworthy note the findings of meta-reviews showing that ‘only in 50% of cases do clinicians follow clinical practice guidelines endorsed by national and professional medical organizations.’ Evans similarly argues that the distinction between normocracy (a focus on rules) and teleocracy (a focus on goals) is a key dividing line in public service management, with rules being the domain of managers and goals the focus of professionals. A similar framing is to suggest that whereas managers value technical rationality, professionals develop and value phronesis – practical wisdom. Phronesis is an expression of both the cognitive and the emotional, which are intertwined in the patient encounter, and is a key element of reflective practice.

A limit to this explanation is that by framing it as a clash between the forms of knowledge favoured by managers and clinicians there is insufficient attention to the ways in which customization bring in a third source of authority: the patient. Conventional accounts of knowledge and evidence (both managerial and professional) become challenged as the authenticity of the user experience is privileged, and forms of informal peer support come to be valued. The new sources of knowledge and claims to expertise have been as challenging to clinicians as they have to managers. For example, an article in *Pulse*, the magazine for general practitioners in the United Kingdom, reported high levels of professional suspicion about the introduction of personal health budgets.^2^ It was headlined, ‘Revealed: NHS funding splashed on holidays, games consoles and summer houses.’ The article went on: “the scheme to give ‘patients more control over their care’ has been used to buy many unevidenced treatments at the expense of long-established services which have been defunded.” The article exemplifies a number of points of professional resistance to personal health budgets, including the way in which patients choose treatments which lack a conventional evidence base.

This understanding then points to a different root to the tension between standardization and customization and calls up a second explanation: it is not a clash between managerial rationality and clinical phronesis but between forms of knowledge which are formalised and replicable and others which are more ad hoc and provisional. Here what is at stake is an epistemological and indeed ontological clash between the modern and the post-modern. Mannion and Exworthy describe standardised processing of patients as McDonaldization (a new take on the older concept of Taylorization). In the call for standardisation (‘each patient/burger to be processed in exactly the same prescribed way’) we see the high point of modernity. In the recognition of diversity, difference, identity, and the validation of patient views as well as doctors, we see the post-modern.

Framing the tension in this way is suggestive of a temporal ordering: the modern (standardization) is being eclipsed by the post-modern (customization). However such a neat ordering is belied by trends in health service management. As the authors point out, standardisation is intensifying its grip in some parts of the health system rather than fading away. Indeed, Mannion and Exworthy suggest that we have not yet reached the high point of the industrialisation of medicine, since we are not yet at the ‘automation’ stage. It is likely that the more fractured and partial insights of postmodernity will continue to co-exist with the claims to efficiency embedded in modernism.

Rather than seeing the tensions between customization and standardization as expressive of an evolution from one era to the next, it may be more satisfactory to reach for a functional explanation: some things that health systems do are amenable to standardised and routinized interventions, whereas others require a more tailored approach. This explanation reminds us of the enormous diversity of tasks and processes undertaken by modern health systems and the unlikelihood of a single logic being appropriate across them all. Hip surgery clearly requires a different set of operating procedures to end of life care, and we can embrace standardization in one whilst rejecting it in the other. However, whilst this works for some bits of health, it quickly becomes clear that the standardised and the customised are much more intertwined that this explanation would suggest. Giving a toddler a Measles-Mumps-Rubella (MMR) vaccine could be seen as emblematic of a standardised procedure, based on a set protocol. But reassuring the parents about the safety of the procedure and calming the child requires improvisatory practices which will need to be personalized to the individual encounter.

The fourth explanation for these tensions, and the one which is best able to account for their enduring presence, is a moral one: standardization and customization relate to differing views of fairness which most of us hold concurrently in a dynamic tension. Recent UK politicians have made political capital out of a rejection of a ‘monolithic’ welfare state based on one size fits all principles, in place of an embrace of personalised care. Mansell and Beadle-Brown suggest, ‘There is now no serious alternative to the principle that services should be tailored to individual needs, circumstances and wants.’^3^ However, the postcode lottery retains its potency as an emblem of unfairness. In health, as in other public services, there is a public and professional ambivalence about simultaneously wanting to uphold sameness and difference.

Attitudes to fairness are not fixed – for example populations are less committed to egalitarian accounts of fairness than they were 50 years ago, and more willing to bring individual responsibility into discussions of equity. Attitudes will also vary by health system and country. Locating the tension between customization and standardization in a moral ambivalence does not mean that the balance between the two will always remain the same. However it does highlight the value of training and supporting staff to understand that ambivalence in their own practice and in the expectations of patients. The legitimacy of a health system depends on its ability to offer the ‘best of both worlds’ to citizens, respecting the reassurance of sameness and the dignity of difference.

## Ethical issues

Not applicable.

## Competing interests

Author declares that she has no competing interests.

## Author’s contribution

CN is the single author of the paper.
